# Endoscopic submucosal dissection of endobronchial leiomyoma with a hybrid knife in an adolescent patient: a case report

**DOI:** 10.3389/fonc.2023.1288044

**Published:** 2023-11-17

**Authors:** Zilin Wang, Gang Hou

**Affiliations:** ^1^ National Center for Respiratory Medicine, State Key Laboratory of Respiratory Health and Multimorbidity, National Clinical Research Center for Respiratory Diseases, Institute of Respiratory Medicine, Chinese Academy of Medical Sciences, Department of Pulmonary and Critical Care Medicine, Center of Respiratory Medicine, China-Japan Friendship Hospital, Beijing, China; ^2^ Peking Union Medical College; Chinese Academy of Medical Sciences, Beijing, China

**Keywords:** endobronchial leiomyomas, hybrid knife, endoscopic submucosal dissection, case report, adolescent

## Abstract

Endobronchial leiomyomas are uncommon benign tracheobronchial tumors. Bronchoscopic intervention is a safe and effective strategy for patients with contraindications for surgery or refusal to undergo surgery. Endoscopic submucosal dissection (ESD) is widely used to treat early gastrointestinal tumors. The novel hybrid knife is useful during ESD owing to functions of submucosal injections, lesion dissection and hemostasis, and makes ESD more convenient. Here, we report a case of a benign leiomyoma at the orifice of RB7 in an adolescent boy. The diagnosis was confirmed based on bronchoscopic and pathological findings. The patient was successfully treated with combined electrocautery snare and cryoresection, ESD using a hybrid knife and the wound was managed with argon plasma coagulation. The postoperative course was satisfactory, with a good general condition and no severe respiratory symptoms. This is, to our knowledge, the first reported case of ESD using a hybrid knife to treat an endobronchial leiomyoma in an adolescent patient.

## Introduction

Endobronchial leiomyomas are rare benign tumors derived from the smooth muscle layer of the bronchi, bronchioles, or blood vessels (1) that account for 3% of all benign endobronchial tumors (2). Considering their rarity, systematic studies on the sex and age distribution, tumor location within the airway, growth patterns, and pathological classifications are lacking. Surgical resection, including sleeve lobectomy, segmentectomy, and pneumonectomy (3), is recommended for endobronchial leiomyomas. Although it often results in favorable long-term outcomes without recurrence, perioperative complications can arise (4). With the development of bronchoscopic techniques, endobronchial interventions have become increasingly viable (5–7). These methods are favored owing to their minimally invasive nature and ease of operation, particularly for benign tumors. Nonetheless, incomplete resection and recurrence after endoscopic approaches, particularly for tumors with a wide base, can occur (4, 8). This underscores the requirement of an endoscopic technique that can completely excise tumors.

Endoscopic submucosal dissection (ESD) is performed for submucosal tumors within the gastrointestinal tract, often achieving curative results (9–13). The hybrid knife, with multifunctional capabilities, such as circumferential dissection, submucosal injections, dissection, and hemostasis (14, 15), has expedited ESD procedures (14). It can also be used to treat recurrent benign tracheal tumors (16). It may represent a novel approach for completely removing benign endobronchial tumors, thereby decreasing recurrence.

Here, we report a case of endobronchial leiomyoma in an adolescent patient treated with ESD using a water-jet hybrid knife.

## Case description

**Figure 1:**
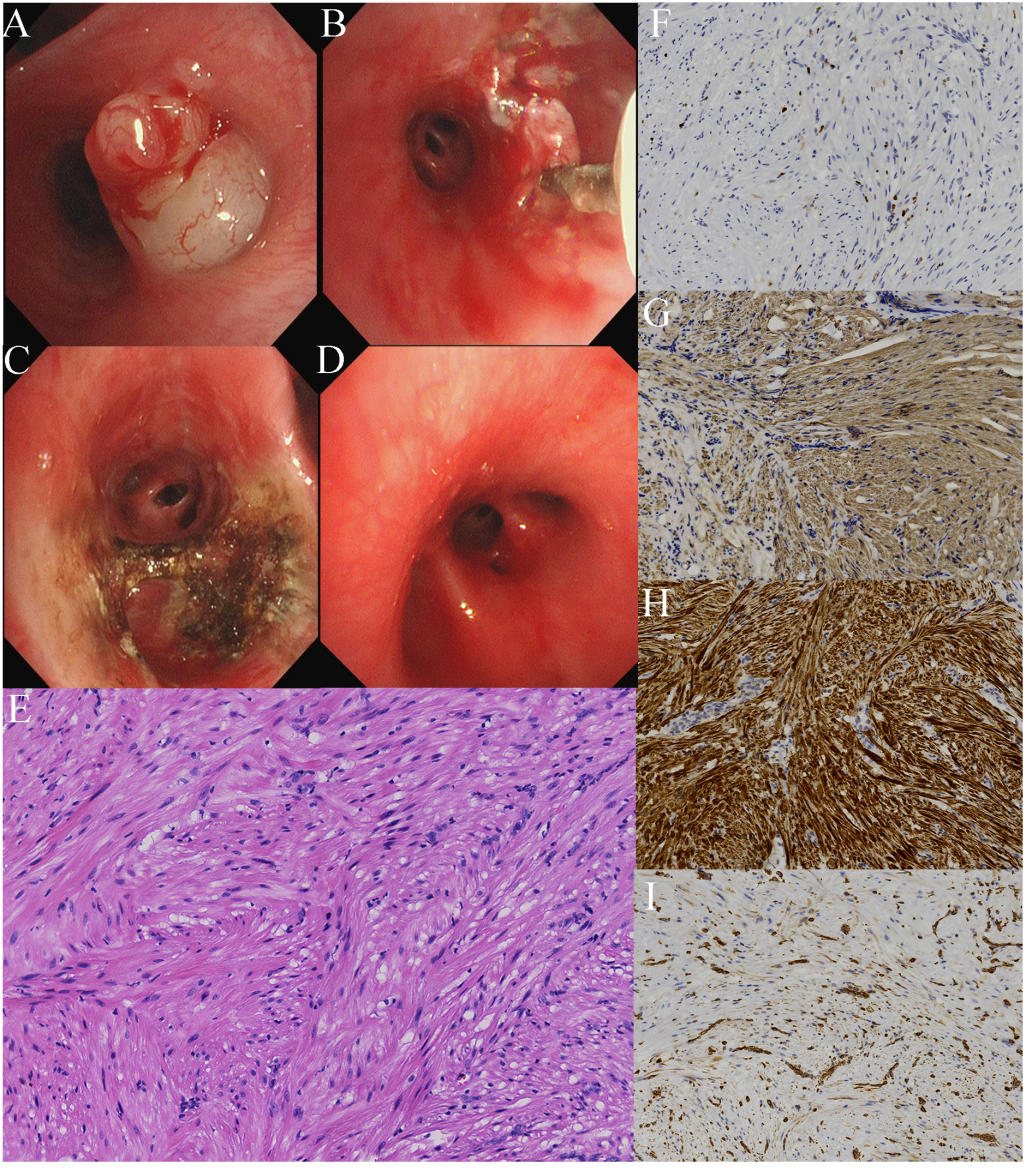
Bronchoscopic examination shows **(A)** an irregular, protuberant mass at the orifice of RB7 on bronchoscopy, **(B)** a hybrid knife used to remove the tumor stump, **(C)** the appearance of the orifice of RB7, after a combined treatment of electrocautery snare, cryoresection, ESD using a hybrid knife, and APC for wound management, and **(D)** the appearance of the orifice of RB7 3-month post-treatment. **(E)** Hematoxylin and eosin staining shows the mass composed of spindle cells. Immunohistochemical staining reveals a **(F)** Ki-67 index of 3%+; the mass is positive for **(G)** alpha-smooth muscle actin, **(H)** desmin, and **(I)** vimentin.

A 16-year-old boy presented to our hospital with a 2-month history of right chest pain and no other respiratory symptoms. Computed tomography conducted at another hospital revealed a high-density nodule-like shadow measuring appropriately 0.6 cm in the bronchial lumen of the right lower lobe. Morphological and immunohistochemical analyses of the biopsied tissue confirmed endobronchial leiomyoma. Although surgical intervention remains the recommended treatment, considering the patient’s age, both the patient and his parents refused a surgical resection. ESD was performed using a water-jet hybrid knife to thoroughly expose the tumor base for comprehensive resection. Under general anesthesia, the bronchoscopy (BF-1T290; Olympus, Tokyo, Japan) conducted using a rigid bronchoscope revealed an irregular, protuberant mass at the orifice of RB7, which obstructed the lumen (**Figure 1A**). Switching to the narrow-spectrum mode highlighted a few blood vessels on the lesion surface. A significant portion of the tumor was excised and collected using an electrocautery snare and cryoprobe. Subsequently, a hybrid knife (HybridKnife^®^ ERBE JET2 Elektromedizin, Tübingen, Germany) was employed to dissect the tumor stump, as described previously (**Figure 1B**) (16). A water jet was used to administer a 2% lidocaine solution into the submucosal layer of the tumor base under a 30-bar pressure. The hybrid knife then entered the submucosal space via the injection site, making a circumferential incision (VIO mode ENDOCUT Q, effect 3-width 1-interval of incision 4, Coagulation effect 2 and 30 W). After the tumor base resection, argon plasma coagulation (APC) was applied to treat the wound surface (**Figure 1C**). Postoperatively, the patient’s general condition was stable, with no signs of chest tightness, chest pain, or hemoptysis. Physical examination revealed no abnormalities. The 3-month and 5-month follow-up showed no signs of recurrence (**Figure 1D**). Long-term surveillance is necessary for further assessment of recurrence.

Hematoxylin and eosin staining indicated that the mass was composed of spindle cells (**Figure 1E**). Immunohistochemical staining revealed a relatively low Ki-67 proliferation index of 3%+ (**Figure 1F**), positive for alpha-smooth muscle actin, desmin, and vimentin (**Figures 1G–I**) and negative for HMB45 and S-100. These pathological results confirmed the diagnosis of an endobronchial leiomyoma.

## Discussion

Benign airway tumors are uncommon, accounting for only 2% of all pulmonary tumors. Endobronchial leiomyomas are extremely rare, accounting for approximately 0.66% of all benign pulmonary tumors (17). These tumors originate from the mesenchymal layer and are composed of bronchial and vascular smooth muscle cells (2). Advanced endoscopic technologies have enabled bronchoscopic interventions as potential alternatives to the endoscopic removal of leiomyomas within the airway. This can be achieved through various methods, such as electrocautery, laser, APC, and cryotherapy (5). Pedunculated benign tumors are often effectively treated using these resection methods, although wide-based tumor recurrence has been reported in a single-institution review (18). Episodes of recurrence have also been reported in tracheobronchial leiomyomas after tumor removal, whether with laser ablation or APC (8, 16, 19). Limited cases and comparative studies may not conclusively prove this, but these instances highlight potential limitations in the width and depth of the current endoscopic approaches (20).

ESD is used to resect superficial gastrointestinal lesions (21). The hybrid knife is a novel tool that integrates a high-pressure water jet with an electrocautery knife. This combination renders ESD more convenient because the same device can be used for submucosal injections, lesion dissection, and hemostasis (14). ESD can effectively expose the tumor base for a more comprehensive resection. Additionally, the submucosal water cushion formed by the hybrid knife acts as a protective barrier for the surrounding tissue and reduces the perforation risk. Previous use of the hybrid knife has shown promising outcomes after the resection of recurrent lesions of benign tracheal tumors in two patients (16, 22). This procedure involves the creation of a submucosal tunnel using a water jet, followed by circumferential dissection of the lesion. The water jet pressure differed between the present case (30-bar pressure) and Gu et al.’s case (40-bar pressure). The choice of a lower pressure in the present case was necessary because of the location of the leiomyoma in the segmental bronchus, which possesses a thinner wall amplifying the perforation risk.

ESD with the hybrid knife is a viable treatment option for endobronchial leiomyoma in adolescents, with no recurrence observed at the 5-month follow-up. However, some limitations should be acknowledged. The evidence was primarily derived from a single case with a 5-month follow-up period. Comprehensive long-term follow-up studies are essential to determine the safety and efficacy of ESD using a hybrid knife in the management of endobronchial leiomyomas. Furthermore, the cost of the hybrid knife and the requirement of specialized equipment may render it inaccessible to some medical facilities.

## Conclusion

In summary, ESD using a hybrid knife is a novel and effective method for treating endobronchial leiomyomas without complications. This approach may emerge as a potential treatment method for benign tracheobronchial tumors. However, considering the relatively short follow-up period and its nascent adoption, a comprehensive evaluation of the safety and efficacy of ESD with a hybrid knife to manage endobronchial leiomyomas requires further clinical research and case studies for validation.

## Data availability statement

The data presented in this study are available on request from the corresponding author.

## Ethics statement

Written informed consent was obtained from the individual(s), and minor(s)’ legal guardian/next of kin, for the publication of any potentially identifiable images or data included in this article.

## Author contributions

GH: Conceptualization, Resources, Supervision, Validation, Writing – review & editing. ZW: Writing – original draft, Writing – review & editing.
